# Unique spectral markers discern recurrent Glioblastoma cells from heterogeneous parent population

**DOI:** 10.1038/srep26538

**Published:** 2016-05-25

**Authors:** Ekjot Kaur, Aditi Sahu, Arti R. Hole, Jacinth Rajendra, Rohan Chaubal, Nilesh Gardi, Amit Dutt, Aliasgar Moiyadi, C. Murali Krishna, Shilpee Dutt

**Affiliations:** 1 Shilpee Dutt Laboratory, Tata Memorial Centre, Advanced Centre for Treatment, Research and Education in Cancer (ACTREC), Kharghar, Navi Mumbai 410210, India; 2 Chilakapati Laboratory, Tata Memorial Centre, Advanced Centre for Treatment, Research and Education in Cancer (ACTREC), Kharghar, Navi Mumbai 410210, India; 3 Integrated Cancer Genomics Laboratory, Tata Memorial Centre, Advanced Centre for Treatment, Research and Education in Cancer (ACTREC), Kharghar, Navi Mumbai 410210, India; 4 Department of Neurosurgery, Tata Memorial Centre, Advanced Centre for Treatment, Research and Education in Cancer (ACTREC), Kharghar, Navi Mumbai 410210, India

## Abstract

An inability to discern resistant cells from bulk tumour cell population contributes to poor prognosis in Glioblastoma. Here, we compared parent and recurrent cells generated from patient derived primary cultures and cell lines to identify their unique molecular hallmarks. Although morphologically similar, parent and recurrent cells from different samples showed variable biological properties like proliferation and radiation resistance. However, total RNA-sequencing revealed transcriptional landscape unique to parent and recurrent populations. These data suggest that global molecular differences but not individual biological phenotype could differentiate parent and recurrent cells. We demonstrate that Raman Spectroscopy a label-free, non-invasive technique, yields global information about biochemical milieu of recurrent and parent cells thus, classifying them into distinct clusters based on Principal-Component-Analysis and Principal-Component-Linear-Discriminant-Analysis. Additionally, higher lipid related spectral peaks were observed in recurrent population. Importantly, Raman spectroscopic analysis could further classify an independent set of naïve primary glioblastoma tumour tissues into non-responder and responder groups. Interestingly, spectral features from the non-responder patient samples show a considerable overlap with the *in-vitro* generated recurrent cells suggesting their similar biological behaviour. This feasibility study necessitates analysis of a larger cohort of naïve primary glioblastoma samples to fully envisage clinical utility of Raman spectroscopy in predicting therapeutic response.

Glioblastoma Grade IV (GBM) is a highly aggressive and malignant tumour, accounting for 50% of all the gliomas^1^,^2^ predominantly occurring in adults. The therapy regime includes maximum debulking of the tumour through surgery, followed by radiation and adjuvant chemotherapy using alkylating agents like temozolomide. However, despite multimodal therapy, almost 90% of the cases recur within 12–15 months of treatment and which/who now become refractory to the multimodal treatment of radio-chemotherapy^3^.

Several factors have been attributed to increased recurrence rate seen in GBM. The presence of cancer cells in the heterogeneous GBM with innate capacity to survive the radio-chemotherapy has been associated with the increased resistance observed in GBM^4^,^5^,^6^,^7^,^8^. Over-expression of proteins like EGFR, Survivin, MGMT and altered metabolic proteins has been reported in these resistant GBM cells^9^,^10^,^11^,^12^. Additionally, the cancer-initiating cells are thought to modulate DNA damage repair proteins including ATM, ATR and MSH6 to impart therapy resistance to GBM. Therefore, the presence of innately resistant cells in the parent tumour has implications in the survival and recurrence of the tumour. The identification of these resistant cells would help in better prognosis of the tumour and optimizing the treatment regimen of patients that may lead to better therapeutic outcomes. However, detection of such resistant sub-population of cells from bulk tumour cells has not been possible using currently available diagnostic techniques.

Raman spectroscopy (RS) is a vibrational spectroscopic technique based on inelastic scattering of light where the energy of photons scattered by the sample is different from the incident photon due to transfer of energy to or from the vibrational modes of molecules in the sample. This technique can be applied on live cells and is sensitive enough to detect subtle biochemical changes in the cells. Because of these reasons, Raman spectroscopy is being extensively explored in the disease diagnosis^13^,^14^,^15^. RS has shown promising results in the diagnosis of several cancers including cervical, lung, oral and brain tumours^16^,^17^,^18^,^19^,^20^,^21^. Most of the studies on brain tumours have focused on *in vivo* and *ex vivo* diagnosis of tumours including gliomas, followed by recent studies on surgical demarcation to determine the precise tumour margins^22^,^23^,^24^,^25^. Recent studies have also shown the utility of Raman spectroscopy and Stimulated Raman Scattering microscopy in detecting the brain regions infiltrated with tumour cells during the course of surgery and distinguishing them from the normal tissue^26^,^27^. The spectroscopic technique has further been used for evaluating the tumour response upon radiation treatment identifying treatment associated changes in tumour^28^,^29^,^30^. Further, RS has been explored for detecting radio-response in cervical cancers, predicting radiation response in 2RT and 5RT tissues^31^ and in oral cancers delving the feasibility of classifying a parental SCC cell line and its radio-resistant 50Gy and 70Gy clones^32^. An exploratory study in predicting recurrence of oral squamous cell carcinoma was also performed on a smaller cohort using serum Raman spectroscopy by our group^33^. Although such remarkable advances in Raman spectroscopy have enabled better tumour detection, Raman spectroscopy has not been explored for detection of the resistant tumour cells from parent population.

In this study, we used recurrent population derived from an *in vitro* radiation model established in our laboratory from primary Grade IV glioma patient samples and cell lines with the aim to explore if the recurrent population can be separated from the parent population on the basis of bio-molecular differences. Here, we first show by biological assays that the recurrent cells are indeed different as they have resistance to radiation and enhanced survival capacity associated with the increased expression of pERK1/2 and Survivin. However, variations in these biological assays were seen in different recurrent populations. We further show that the whole transcriptome analysis invariably identified two different transcriptional landscapes of the parent and recurrent population of cells. Since detection of these resistant populations required a global means of detection, we demonstrate the efficiency of Raman spectroscopy, a non-invasive technique that can identify subtle biochemical variations, in differentiating naïve parent and recurrent populations. The data reveals that the Raman spectroscopy can classify the recurrent population into a cluster distinct from parent population. Spectral profiles demonstrate increase in lipid and an overall shift from protein to lipid as hallmarks of the recurrent population. The potential of Raman spectroscopy was then evaluated on an independent set of primary human GBM tissues wherein the efficacy of RS in classifying samples differing in their clinical outcome: responders and non-responders were investigated. Principal Component Analysis (PCA) and Principal Component-Linear Discriminant Analysis (PCA-LDA) revealed separate clusters corresponding to the responding and non-responding patient samples. The spectral profiles identified modulation of lipid along with an additional DNA-related features in the tissues from non-responders compared to the responders. Prospectively, studies on larger cohort of the GBM tissues will be required to validate findings of this preliminary study before implicating Raman spectroscopy in the prediction of GBM patient’s outcome.

## Results

### An *in vitro* radiation resistant model derived from patient samples and cell lines shows the formation of recurrent population upon exposure to lethal dose of radiation

**Figure 1:**
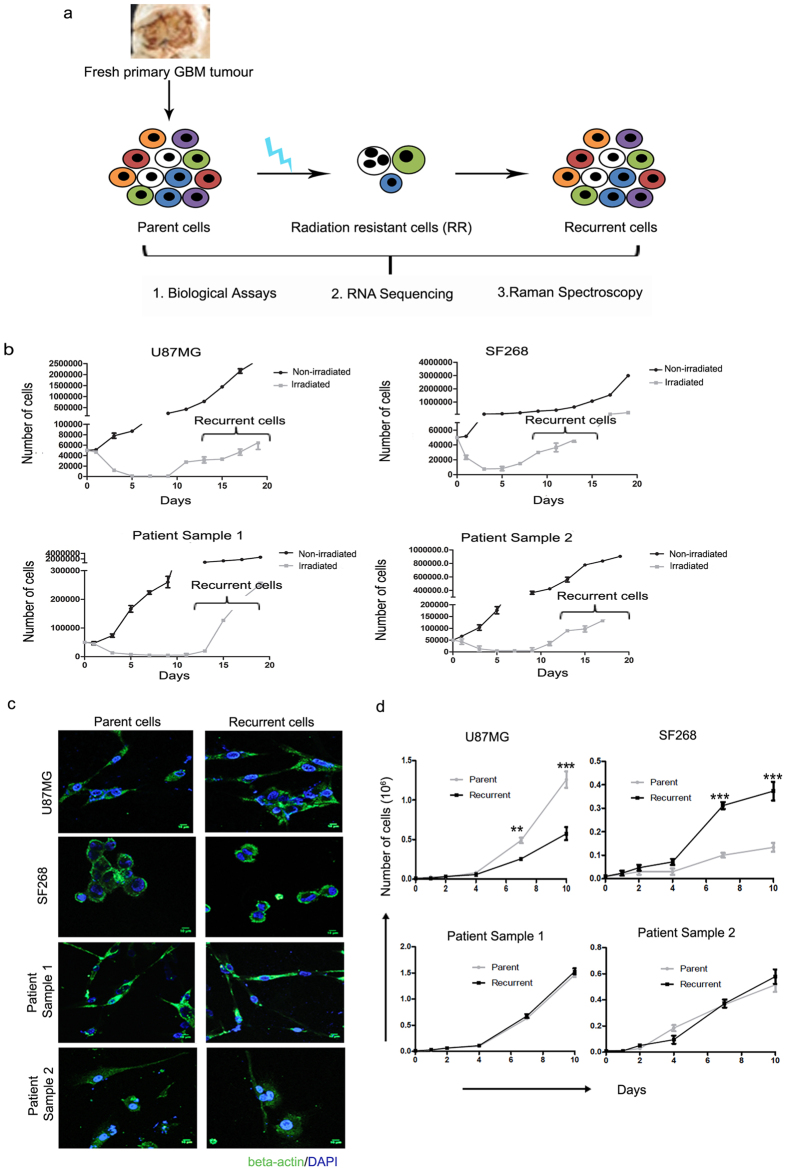
Recurrent cells generated from cellular model shows similar morphology and growth rate as that of parent cells. (**a**) Shows schema of the study. (**b**) Graph shows the growth pattern of non-irradiated and irradiated parent cells from U87MG, SF268, patient sample 1 and patient sample 2. (**c**) Immunofluorescence images of the parent and the recurrent cells from the two cell lines and patient samples as indicated. Cells are stained for β-actin (green) and counterstained with DAPI (blue) to visualize the nucleus. (**d**) Line graphs show the growth curve for parent and recurrent cells. Cell growth was monitored by trypan blue assay. Results in each graph are the composite data from three independent experiments performed in triplicate (mean ± SEM); * denotes p ≤ 0.05, and ** denotes p ≤ 0.01. Scale bar for immunofluorescence images- 10 μm.

The presence of innately resistant GBM cells has long been associated with therapy refraction seen in GBM. With the aim to understand the molecular mechanisms responsible for resistance of GBM cells to radiation, we used a cellular radiation resistant model developed in our lab from the primary GBM patient samples and cell lines^34^. Briefly, we first established primary cultures from naïve Glioblastoma patient samples and characterized for the presence of p53 and ATRX as well as IDH1 mutation (Supplementary Figure 1). Post treatment of parent cells derived from these primary cultures with lethal dose of radiation, a small population of cells survived (after approximately a week of radiation) termed as innately radiation resistant (RR) cells which displayed a reversible senescent phenotype. As shown in Fig. 1a, these early phase RR cells eventually underwent atypical cytokinesis to form recurrent tumour population (R) 15–22 days post radiation. Recurrent cells thus obtained were employed for biological and Raman spectroscopic analysis^34^. Importantly, similar results are obtained when cells are subjected to clinically pertinent fractionated doses of radiation. Here, we have used two cell lines U87MG and SF268 and two primary cultures established from Glioblastoma grade IV patient samples. For all the experiments performed in this study, individual cultures were treated with their respective lethal dose of radiation (as identified previously using clonogenic assays) and cultured to obtain the recurrent population generated after about 15 days of radiation treatment (Fig. 1b). Three independent batches of recurrent cells were generated (biological triplicates) from each sample and used for further analysis as shown in the scheme (Fig. 1a).

### Recurrent cells have similar morphology and proliferation rate as compared to the parent population

In the first step, morphological characteristics of the parent cells were compared with its recurrent cell counterpart to explore the presence of any visually apparent differences. Cellular morphology of parent and recurrent cells from all cultures was visualized by fluorescence microscopy after staining for β-actin, a cytoskeletal protein. As shown in Fig. 1c, no substantial differences between the morphological features of the two populations were observed. Further, proliferation potential of these cells was analysed using trypan blue assay for 10 days. Variability was observed in case of the cell lines; recurrent cells from U87MG cell line showed lower proliferation rate whereas SF268 recurrent cells showed higher rate of proliferation as compared to the parent population. The recurrent cells formed from the primary cultures of patient samples did not show any enhancement in the growth potential as compared to their parent cells (Fig. 1d). This suggests that recurrence of the glioma cells does not depend entirely on increased cellular proliferation. Indeed, Schröder *et al*. showed that the proliferation index remained similar in both the recurrent and primary tumours^35^.

### Recurrent cells possess enhanced radiation resistance as compared to the parent population

**Figure 2:**
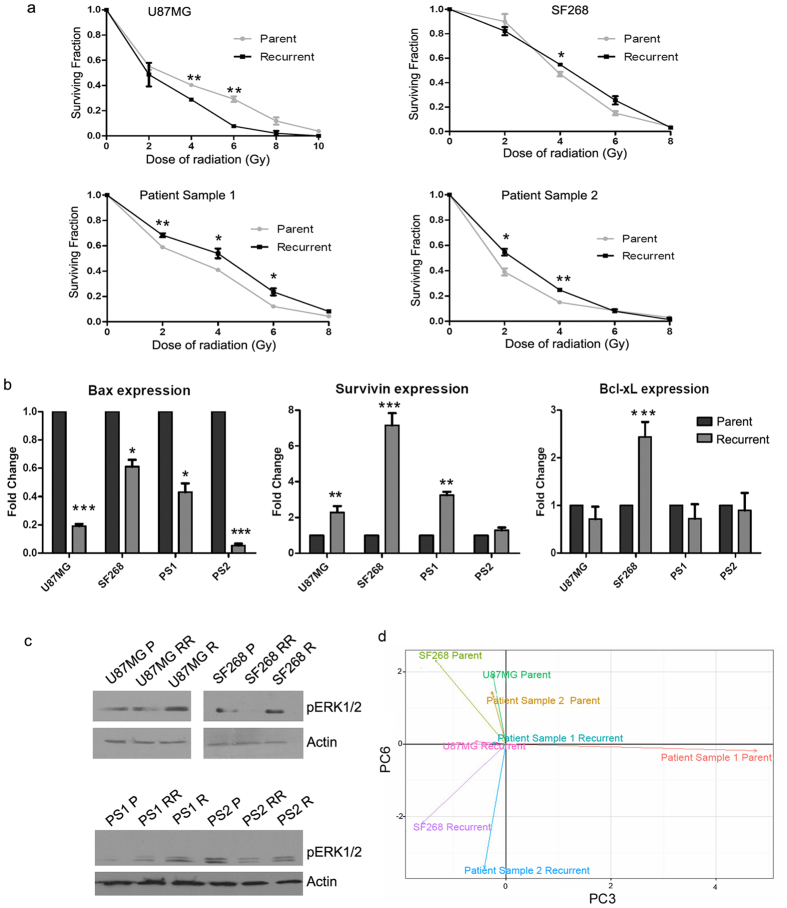
Recurrent cells reveal differences as compared to parent cells at the molecular level. (**a**) Clonogenic survival assay curve showing the survival fraction at different doses of radiation for parent and recurrent cells of U87MG, SF268, patient samples 1 and patient sample 2. (**b**) Bar graph shows the transcript levels of pro-survival genes Survivin and Bcl-xL and pro-apoptotic gene Bax in parent and recurrent population as determined by qPCR. (**c**) Western blot analysis using anti-pERK1/2 and β-actin antibodies on parent cells (P), radiation resistant (RR) and recurrent (R) cells of the indicated samples. PS1 represents patient sample 1 and PS2 represents patient sample 2. (**d**) PCA plot of global transcripts between parent and recurrent cells. Results in each graph are the composite data from three independent experiments performed in triplicate (mean ± SEM); *** denotes p ≤ 0.001.

Since the recurrent cells are formed from the innately radio-resistant cells, we examined the resistance potential of the recurrent and parent cells. As shown in Fig. 2a, clonogenic assay was performed to compare the survival of the parent and the recurrent cells at different doses of radiation. We observed that the recurrent cells from the patient samples as well as SF268 indeed had significantly higher cell survival at the low doses of radiation ranging from 2–6 Gy (Fig. 2a). The D_0_ (dose at which 37% of cells survive upon radiation treatment) of the recurrent cells was found to be 4.1, 5.2, 4.7 and 4.2 Gy whereas it was found to be 5.79, 4.79, 3.5 and 3.4 Gy in the parent population of U87MG, SF268, patient sample 1 and patient sample 2, respectively. These data show the enhanced radio-resistant character of the recurrent cells generated from the patient samples and SF268 cell line. However, the U87MG recurrent cells showed higher radio-sensitivity as compared to the parent cells (Fig. 2a).

### Recurrent cells show up-regulation of survival pathways

Enhanced survival capacity is a known property of treatment-resistant recurrent GBM cells. Therefore, the transcript levels of known pro-survival genes Survivin, Bcl-xL and pro-apoptotic gene Bax in the recurrent and parent cells were investigated by quantitative PCR (Fig. 2b). The graph shows that recurrent cells had lower mRNA expression of pro-apoptotic gene Bax when compared to their parental counterpart whereas the transcript levels of Survivin was 2–7 fold higher in these cells compared to the parent cells. However, patient sample 2 (PS2) showed only a marginal increase in the Survivin expression. The increased expression of Survivin in the recurrent cells formed upon lethal exposure of radiation is noteworthy since Survivin has been shown to be overexpressed in Glioblastoma tumours associated with high anti-apoptotic activity^36^,^37^. The expression of other pro-survival gene Bcl-xL in the recurrent cells remain unaltered except for SF268 cells (Fig. 2b), may be due to the dependency of GBM recurrent cells on Survivin for its anti-apoptotic activity. MAPK pathway- another major survival pathway known to promote tumour growth was also investigated for the phosphorylation of ERK1/2 in the parent, immediately after IR in the RR cells and recurrent (R) cells. It was observed that as compared to parent cells, recurrent cells had higher expression of pERK1/2 indicative of heightened survival capacity in recurrent cells, except in the PS2 recurrent cells where the levels of pERK1/2 were comparable to parent cells (Fig. 2c). The expression of pERK1/2 was less pronounced in the cells generated immediately after radiation as compared to the recurrent cells (R) probably due to late activation of survival signals.

### Whole transcriptome analysis confirms recurrent cells are different from parent cells

As opposed to a particular biological behaviour where fewer molecular players impart that specific characteristic to a cell, whole transcriptome analysis provides a global picture of cellular transcriptional activity at any given time. Hence, we performed whole transcriptome sequencing of the parent and recurrent cells. Principal Component Analysis (PCA) of the transcriptome data showed Principal Components 3 and 6 captured maximum variation from the data, distinguishing the recurrent cells from parent cells, while patient sample 1 parent clustered separately (Fig. 2d).

Collectively, these data show that the parent and recurrent populations were morphologically similar but differing in certain biological properties like proliferation and radiation resistance. Because of the inconsistencies observed in these properties among different populations, neither of them can be used as a hallmark of either parent or recurrent population (Fig. 2a–c). However, global expression profile of these samples by RNA-sequencing revealed unique transcriptional landscape for these populations, separating them into distinct clusters. This result suggested that the use of methods that detect global molecular changes in parent and recurrent cells could uniquely identify parent and recurrent cell populations as separate entities. Thus, we explored Raman spectroscopy, a rapid, label-free, cost-effective, non-invasive approach which yields global or holistic information about the biochemical milieu of the sample and may provide unique spectral markers for recurrent cells.

### Mean and difference spectra from Raman Spectroscopy of the parent and recurrent population reveals spectral features unique to recurrent population

**Figure 3:**
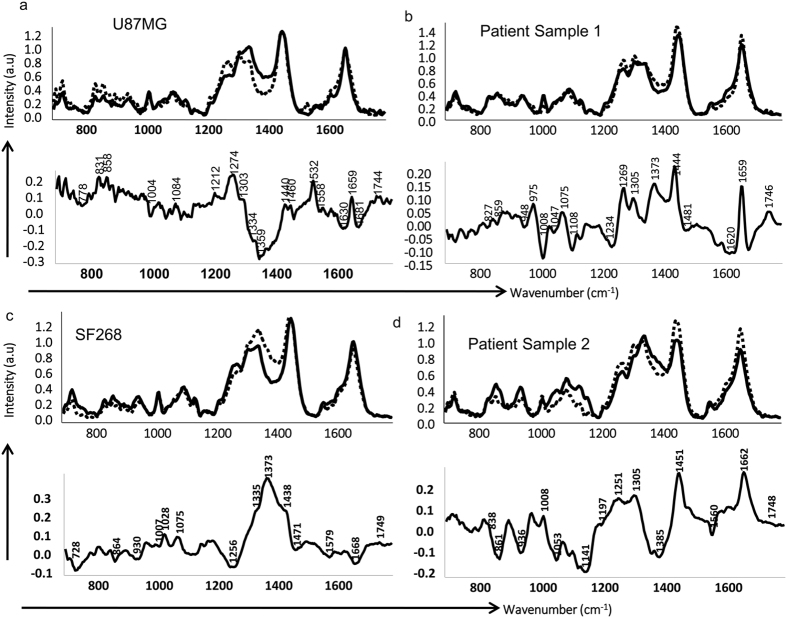
Mean and difference spectra from parent and recurrent population reveals spectral features unique to recurrent population. (**a–d**) Shows the mean spectra and difference spectra of parent and recurrent population obtained for U87MG, SF268, patient samples 1 and patient sample 2, respectively. Dotted lines represent recurrent cells and solid lines represent parent cells.

Raman spectroscopy of parent and recurrent cell populations of all the cultures was carried out as described in the materials and methods section. First, mean spectral comparisons were undertaken, followed by multivariate analysis of the data. The mean normalized spectra for parent and the recurrent cell lines from both the patient samples and cell lines were computed for both fingerprint (700–1800 cm^−1^) as depicted in Fig. 3 and high wavenumber (2800–3100 cm^−1^) regions as shown in Supplementary Figure 2. Average spectra from each parent population were overlaid with their respective recurrent population for better comparison and understanding. Additionally, difference spectra between parent and recurrent population were also generated as shown in Fig. 3a–d and Supplementary Figure 2a–d and annotated based on previous reports^38^,^39^,^40^,^41^,^42^. The analysis of the spectra showed that features corresponding to higher DNA content (1095 cm^−1^- DNA backbone, 1340 cm^−1^-total nucleic acid content and 1610 cm^−1^-cytosine base), protein related features like amide III (1260 cm^−1^), CH_2_ bending at 1450 cm^−1^, phenylalanine (1008 cm^−1^), tryptophan (1560 cm^−1^) and sharp features around amide I (1660 cm^−1^) characterized the parent populations. Thus, in parent population, higher DNA content and overall protein-related features were observed. On the other hand, prominent features of recurrent population included lipid-related features like 1272 cm^−1^ and 1305 cm^−1^, sharp 1447 cm^−1^,1725 cm^−1^ and 1746 cm^−1^; protein bands like amide III (1262 cm^−1^), shifted CH_2_ bend (1447 cm^−1^) and sharp amide I (1660 cm^−1^). The band at 1660 cm^−1^ could also be attributed to ceramide backbone as another minor shoulder band was observed at 1673 cm^−1^ in some recurrent cells’ spectra. Difference spectra were also computed by subtracting parent cells’ spectra from recurrent cells’ spectra which showed positive peaks at 1260–70 cm^−1^, 1305 cm^−1^, 1440–50 cm^−1^, 1660 cm^−1^and 1744 cm^−1^ corresponding to lipid/phospholipid and protein content and negative bands at 1075–1090 cm^−1^, 1330–40 cm^−1^, 1480 cm^−1^ corresponding to DNA (Fig. 3a–d). However, SF268 recurrent population was characterized by lower protein and slightly higher DNA content compared to the parent cells. Mean spectral analysis in high wavenumber region depicted in Supplementary Figure 2a–d indicated shoulder band in the 2850–2900 cm^−1^ region and band in the region of 2900–2950 cm^−1^. In all the samples, a higher breadth and intensity in the 2840–2880 cm^−1^ region corresponding to lipid content was observed in the recurrent samples with respect to parent samples while positive peak around 2900 cm^−1^ indicative of protein was seen in parent cells. Thus, from parent to recurrent, in both the fingerprint and high wavenumber regions, a shift towards increased lipid content was observed. This shift from protein to lipid synthesis in the recurrent population is consistent with several reports, demonstrating an increase in the lipid biogenesis contributing to resistance to chemotherapy and radiotherapy^43^,^44^,^45^. Also, a sharp peak observed at 1660 cm^−1^ in the recurrent population attributed to ceramide has been implicated in driving cancer resistance to various chemotherapeutic drugs^46^,^47^ and may play a role in promoting radio-resistance. Thus, Raman spectroscopy analysis revealed characteristic features for both recurrent and parent cells in terms of peak position and intensity variations. Major features observed in recurrent cells included lower DNA content, higher lipids, phospholipids and proteins.

### Principal component analysis (PCA) identifies unique clustering of recurrent and parent cells

**Figure 4:**
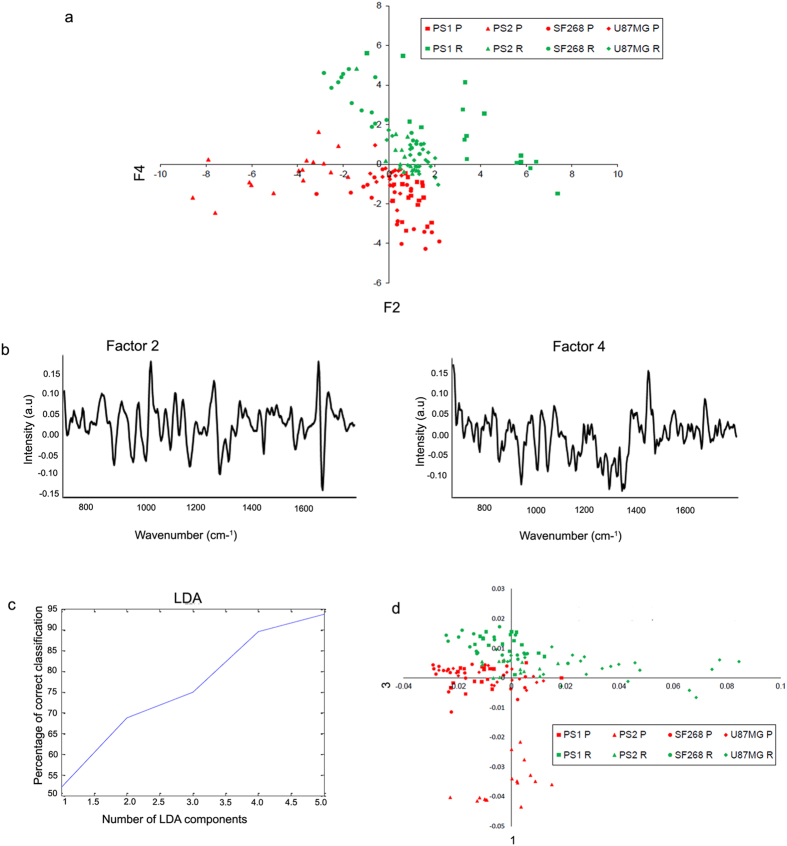
PCA and PCA-LDA analysis classifies the recurrent cells from parent cells. (**a**) PCA scatter plot for parent and recurrent populations from the cell lines and two samples. (**b**) Spectra show the loadings of factor 2 and 4. (**c**) The PCA factors used for LDA are shown. (**d**) PCA-LDA scatter plot for parent and recurrent populations are shown.

As the parent and recurrent cells showed variations in their Raman spectra, the feasibility of classification of parent and recurrent population as distinct entities using RS was also explored. First, an unsupervised Principal component analysis (PCA) was performed to obtain a unique classification of the recurrent population (obtained from primary cultures and cell lines) as compared to their parent counterpart. PCA was carried out using 10 factors. Out of these, maximum variability could be captured between factor 2 and 4. Scatter plot between factor 2 and 4 indicated classification between each of the parent and recurrent population (Fig. 4a). In fact, two almost distinct clusters corresponding to parent and recurrent population were observed. The loadings spectra corresponding to factors 2 and 4 are shown Fig. 4b. The loadings of factor 2 showed spectral features corresponding to DNA (1072 cm^−1^, 1345 cm^−1^, 1380 cm^−1^), and proteins (1255 cm^−1^,1448 cm^−1^, 1665 cm^−1^, tryptophan 1559 cm^−1^) while factor loading 4 had features from lipids (1440 cm^−1^, 1740 cm^−1^), DNA (1072 cm^−1^, 1344 cm^−1^, 1373 cm^−1^) and proteins (1250 cm^−1^, 1650 cm^−1^, phenylalanine (1005 cm^−1^). PCA for high wavenumber region (2800–3100 cm^−1^) was also carried out and showed a tendency of classification between parent and recurrent spectra of all samples (data not shown). Although PCA is a data visualization tool, this analysis indicated that the unique pattern clustering is due to overall differences in the biochemical profile of recurrent and parent cells. The data also revealed that exposure to radiation alters the biochemical profile of a cell.

### Principal component-linear discriminant analysis (PC-LDA) distinctly classifies the recurrent cells from parent cells

Upon obtaining modest classification of all recurrent cells from parent population using PCA, a method used for exploratory data analysis unravelling trends and outliers in the data, PC-LDA, a supervised method of data analysis, was explored for better classification. In the first step, standard models using one set of experimental data from parent and recurrent populations of U87MG, SF268, patient sample 1 (PS1) and patient sample 2 (PS2) were built and validated by leave-one-out-cross-validation (LOOCV) In the subsequent step, the model was evaluated by independent data acquired from PS2 and U87MG cell line.

Five factors accounting for ~93% correct classification were used to build the standard model (Fig. 4c). The scatter plot for PC-LDA is shown in Fig. 4d. Like PCA, two major clusters representing all parent and all recurrent populations were observed. As shown in Table 1, almost 100% classification efficiency was achieved for all parent and recurrent populations, indicating important and distinct differences between these two populations. Moreover, even in LOOCV, >95% classification efficiency was observed for all groups with minor misclassifications between recurrent populations of U87MG, SF268 and PS1. Upon test prediction on this standard model, 16/16 spectra from PS2 parent group and 14/16 spectra from recurrent group were correctly classified. All 18 spectra from U87MG parent group were correctly classified and 16/22 recurrent population could be correctly classified. Of the 6 misclassifications in U87MG recurrent population, 5 were classified as PS2 recurrent group indicating overlapping features between these two samples. Thus, the efficacy of RS in correct identification of parent and recurrent populations was demonstrated.

### Raman spectral analysis of GBM tissues classified a population of GBM tissues differing in clinical outcome

**Figure 5:**
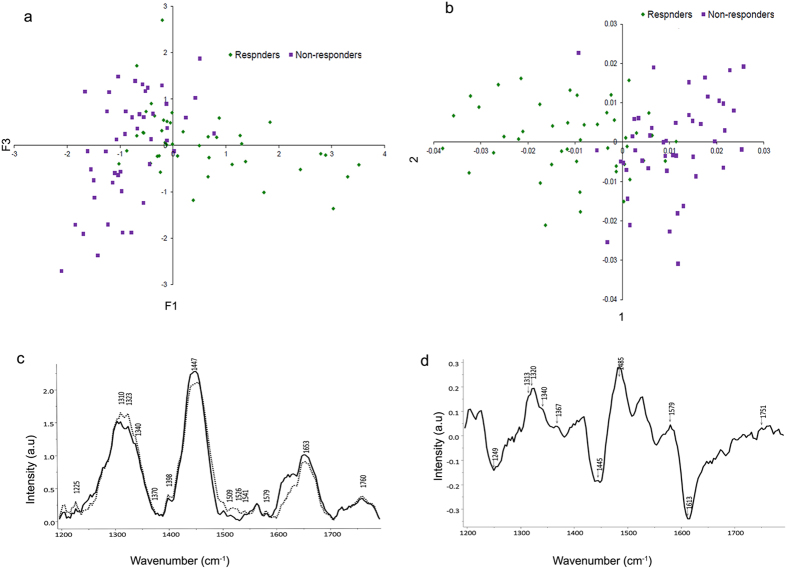
Raman spectroscopic analysis distinctly classifies the non-responders from responders group of patients. (**a**) PCA scatter plot for non-responders and responders patient samples. (**b**) PCA-LDA scatter plot for non-responders and responders patient samples. (**c**) Mean spectra of the responding group from the non-responding group of samples. Dotted lines represent non-responding group while solid lines represent responding group of patients. (**d**) Difference spectra computed after subtracting spectra of responding group from the non-responding group of samples.

As shown above, we could demonstrate that Raman spectroscopy was able to distinguish between biologically variable recurrent cells from their parental counterpart. However, as patient derived primary cultures do not exactly recapitulate the clinical samples, we further went on to examine independently the feasibility of Raman spectroscopy in categorizing the highly heterogeneous primary tissue samples. As previously reported, presence of innately resistant cells within GBM tumours affect overall survival of the patients^7^,^8^. Therefore, we hypothesized that patients responding to standard treatment (responders) may have lower or no percentage of innately resistant sub-population while the patients non-responsive to standard treatment (non-responders) may have higher percentage of these innately resistant cells. Based on this hypothesis, 6 GBM primary tissues with confirmed clinical outcome (responders = 3 and non-responders = 3) were analysed in this preliminary study to investigate if tumours with differential treatment response can be distinguished using RS. PCA and PCA-LDA of these tissues identified two almost distinct clusters belonging to responders and non-responders group with minor overlap (Fig. 5a,b, Table 2). Average spectra analysis was also undertaken to understand the bio-molecular basis of classification. Spectral features for responders and non-responder tissues indicate prominent features of protein (amide III, CH_2_ deformation, C = C, Tyr, Trp and amide I), DNA (1320, 1340, 1485 cm^−1^) and lipids (1313, 1750 cm^−1^) as shown in Fig. 5c. To understand the changes pertaining to non-responders group, difference spectra were computed. The non-responders difference spectra identified positive spectral features at 1313 cm^−1^ (CH_3_/CH_2_ twisting/bending/wagging of lipids; CH_3_CH_2_ twisting mode of lipid/collagen), 1320–1321 cm^−1^ (DNA bases, Amide III (alpha-helix), CH_2_ deformation of lipids), 1340 cm^−1^ (nucleic acid content), 1367 cm^−1^ (phospholipids), 1485 cm^−1^ (G and A bases of DNA), 1579 cm^−1^ (DNA and heme) and 1750 cm^−1^ (lipid) in the non-responding group (Fig. 5d). Interestingly, some spectral similarities were observed in lipid related features between non-responding group and the recurrent samples from our cell cultures. However, biological meaning of this similarity requires further investigation. Additionally, spectral features of DNA also contributed to their individual classification. Overall these results indicate that Raman spectroscopic analysis of the highly heterogeneous primary tissues obtained before any chemo-radiation therapy may help in predicting prognosis of the patients.

## Discussion

Glioblastoma is a highly lethal type of brain tumour with less than 5% 5-year survival rates^48^. Even after extensive studies on GBM, the prognostic determinants have been limited to the methylation status of MGMT, mutations in IDH1, PTEN and Karnofsky performance score (KPS)^49^. RS is a non-invasive technique that provides insights into the chemical milieu of the samples. Apart from its application in the detection and classification of malignant cells from the normal cells, RS has also shown potential in predicting the radiation response from the tissues and recurrence from serum samples^19^,^26^,^28^,^29^,^30^,^32^,^33^,^50^. A recent development of hand held Raman probe has enabled the detection of brain tumour cells with higher efficiency during the surgery^27^.

These studies have so far investigated diagnosis and surgical demarcation in glioma. As radiation resistance is the primary cause of poor survival rates in glioma patients, early detection of these tumours can possibly help in optimizing the treatment regime and help in improving prognosis of these patients. To understand the mechanisms responsible for radiation resistance, a cellular radiation resistance model was developed previously in our lab^34^. Most of the recurrent populations generated in this model displayed higher survival capacity at low dose of radiation mediated by enhanced pERK1/2, higher mRNA expression of Survivin and down-regulation of Bax as compared to the naïve parent cells. Balance between the pro-apoptotic and pro-survival genes are known to determine the fate of the cells, with over-expression of pro-survival genes imparting resistance phenotype to the recurrent cells. Additionally, whole transcriptome analysis classified the recurrent samples separately from the parent cells, however; intra sample heterogeneity was also seen as it is the inherent property of GBM cells.

As an alternative, Raman spectroscopy- a sensitive technique based on vibrational spectroscopy known to provide holistic information about the biochemical changes inherent to the sample, was evaluated for the detection of these recurrent cells. Using Raman Spectroscopy, we were able to distinguish the recurrent cells from the parent populations of primary patient cultures and cell lines. Raman spectral features in the recurrent cells revealed significantly different biological composition seen in lipid, DNA and protein content of these cells. Variations were also seen in the spectral features of individual recurrent and patient samples in the form of minor spectral shifts and intensity-related differences. These inter-sample differences apparent between parent (or recurrent) populations from different origins were however, less significant than spectral features characteristic to parent and recurrent cells. PCA and PCA-LDA highlighted these global features specific to both parent and recurrent cells and brought about classification between parent and recurrent populations from different samples. However, variation was seen in the recurrent cells from SF268 which revealed higher DNA content and lower protein content as compared to the parent cells. This cell line also demonstrated atypical behaviour in different biological assays; this unusual biological behaviour of SF268 cell line could be the basis for the observed findings. The variability observed with respect to SF268 cell line may be reflective of the heterogeneity existing in GBM and may lead to a reduced sensitivity of any analytical method aimed at detecting recurrent cells.

GBM tumours are known to be highly heterogeneous and the inherently recurrent cells may vary in different tissues. Our previous reports with cell-based Raman spectroscopic studies have examined the feasibility of differentiating normal and abnormal (pre-malignant or malignant phenotype) cells in both oral and cervical cancers^51^,^52^ and also the feasibility of identifying a cancer cell in a mixed cell population having subtle variations has been demonstrated^53^. In case of oral and cervical cancers, heterogeneous cell populations were obtained on exfoliation. In most subjects, the atypical and malignant cells were obscured by presence of overwhelmingly large number of normal cells. However, using a pellet-based approach, RS was able to identify the small number of abnormal cells among the heterogeneous group of samples with ~80% efficiency. Additionally, the classification and characterization of cancer cells exhibiting MDR phenotype has also been demonstrated in the sarcoma cell lines using RS^54^. RS analysis on primary GBM tissues was also conducted in this study to investigate the feasibility of RS to classify these primary tissues based on their clinical outcome (responders or non-responders to treatment). PCA and PCA-LDA classified these tissues into two groups with minor overlap observed amongst them, contributed by the common clonal cells. Spectral analysis revealed the presence of higher lipid and DNA related features in the non-responding group of patients compared to the responding group. Spectral similarity between the non-responders and the *in vitro* recurrent cells was observed but requires further examination for a meaningful interpretation of this observation. Additional features unique to either cells in culture or primary tumour samples were also seen, however a direct correlation between the two was not envisaged in this study. Further, several other spectral features characteristic to non-responders in the GBM tissue study could be attributed to increased spot size, tissue architectural and morphological contributions, including deeper areas attributed to a penetration depth of ~5 mm using the fibre-probe based system.

Our preliminary findings indicate potential of RS in identifying recurrent cells separately from the parent cells using cellular resistance model. Since cell line based model systems do not adequately represent the heterogeneous GBM disease, we examined Raman spectra of an independent cohort of tumour samples where we found that RS could classify these tissues based on their therapy response. Nevertheless, extensive studies on larger cohort of primary GBM patients before any radio-chemotherapy intervention need to be undertaken to confirm the present findings. These studies can then set the stage for clinical translation of Raman spectroscopy for glioblastoma prognostics.

## Materials and Methods

All the methods carried out in this study were in accordance with the approved guidelines and regulations.

### Cell culture

Glioblastoma Grade IV cell lines U87MG and SF268 were obtained from ATCC in 2011. These cell lines were last authenticated in the laboratory by Short Tandem Repeat (STR) profiling based on eight markers in August 2014. The cell lines were grown and maintained in DMEM containing 10% (v/v) fetal bovine serum (Gibco), penicillin (200 U/mL), streptomycin (100 μg/mL) and incubated at 37 °C at 50 mL/L CO_2_ for all the experiments.

### Establishment of short term primary cultures

The project and the experiments on human tumour samples were approved by the Tata Memorial Centre institutional ethics committee (TMC-IEC III) and a written informed consent in the language understood by the patients was also taken prior to tumour collection. Short term primary cultures were derived from fresh Glioblastoma grade IV tissue samples using gentleMACS dissociator after the removal of necrotic tissue. Cell suspension of tumour was seeded and maintained in DMEM: F12 media containing 15% (v/v) fetal bovine serum (Gibco), 1% of antibiotic cocktail containing fungizone and incubated at 37 °C in a humidified incubator with an atmosphere of 5% CO_2_.

Six histopathologically confirmed GBM primary tumours were also collected after patient’s consent and frozen at −80 °C until the acquisition using Raman spectroscopy. All the patients underwent complete total resection followed by standard radio-chemotherapy. The clinical information was obtained from their medical records or by the telephonic correspondence. The survival patients with no recurrence or disease progression even after 3 years of follow up were included in the responders group. Patients with tumour-associated mortality within the study period were included in the non-responders group.

For establishment of radio-resistant populations, samples were irradiated using ^60^Co-γ Bhabhatron-2 radiator (ACTREC, Tata Memorial Centre). Three independent batches of recurrent population were derived from parent population of two patient samples (PS1 and PS2) and two cell lines U87MG, SF268 and were assessed using different biological assays and Raman spectroscopy.

### Characterization of the *in-vitro* radiation model

#### Growth curve

To monitor the cell survival post radiation, two million cells from non-radiated and radiated cultures were irradiated with their respective lethal dose of radiation. Viable cells from these plates were counted every alternative day till 22 days on a hemocytometer using trypan blue dye. Additionally, growth potential of recurrent cells as compared to parent cells was also determined using trypan blue method.

#### Immunofluorescence

To examine the morphological changes, parent and recurrent cells on cover slips were fixed with methanol: acetic acid (2:1) at −20 °C for 10 minutes then washed with 1X PBS and permeabilized with 0.5% Triton X-100 for 15 minutes on ice. After subsequent washing, the cells were incubated for one hour at 37 °C in 5% BSA solution (Sigma Aldrich), followed by overnight incubation with β-actin (mouse; 1:500; Sigma Aldrich) and ATRX (rabbit; 1:750; Abcam) at 4 °C. After washing thrice for 5 min with PBS, coverslips were incubated with FITC conjugated goat anti- mouse antibody (1:100; Cell Signalling) for 45 min. Nuclei were counterstained with DAPI (0.5 μg/mL) for 1 minute, washed thrice with 1X PBS and mounted using VECTASHIELD mounting media (Vector Labs). The cells were visualized under Zeiss LSM 510 Meta Confocal Microscope.

#### Clonogenic survival assay

In order to determine the radio-resistance potential of both parent and recurrent cells, cells seeded in 60 mm dishes were irradiated with different doses of radiation. The cells were then incubated for 11–18 days till the colonies appeared. Colonies were fixed with pre-chilled methanol: acetic acid (3:1) stained with 0.5% crystal violet and counted under the microscope to determine percentage survival.

#### RNA extraction, cDNA synthesis and qPCR

The expression levels of genes Survivin, Bcl-xL and Bax were determined using qPCR. For this, total RNA from parent and recurrent population was extracted by TRIZOL Reagent (Invitrogen) according to the manufacturer’s protocol. cDNA was synthesized using the SuperScript III First-Strand kit (Invitrogen) as per the manual instructions. qPCR was carried out using Roche Light Cycler Master Mix using Light Cycler 480 real time PCR system. GAPDH was used as an internal control. Relative changes of mRNA amounts were calculated using the ΔΔCt method.

#### Western blot analysis

Cell were harvested and lysed for 45 minutes on ice using NP-40 lysis buffer containing 120 mM NaCl, 50 mM Tris-Cl (pH 8.0), 0.5% (v/v) Nonidet P-40, 50 μg/ml PMSF and protease, phosphatase inhibitor cocktail. The supernatant containing the cytoplasmic proteins were quantified by Bradford assay. 30 μg of protein was loaded onto denatured SDS-PAGE, immuno-blotted using anti-pERK1/2 (1:1000; Cell Signalling 4370S), total p53 (1:1000; Cell Signalling 1C12) and β-actin (1:5000, Sigma A5316). The immune-reactive proteins were then visualized using enhanced chemiluminescence (ECL) reagent (Pierce).

#### PCR and Sanger sequencing of IDH1

Genomic DNA was extracted from primary cultures using Qiagen QIAmp DNA Mini kit. Exon 4 of IDH1 was amplified with 100 ng of genomic DNA using following primers:

Forward: CGGTCTTCAGAGAAGCCATT; Reverse: GCAAAATCACATTATTGCCAAC.

The PCR product (2.5 ng) purified using the Qiaquick PCR purification kit was used along with 1.5 pmols of the forward or reverse primer was used for sequencing in the Applied Biosystems DNA Analyser. Mutation Surveyor Software was used to identify mutation in these samples.

#### Whole Transcriptome sequencing analysis

Total mRNA from recurrent and parent populations of two patient samples (PS1 and PS2) and two cell lines U87MG and SF268 were extracted (Dynabeads, mRNA Direct Micro Kit, Invitrogen), used for library preparation and sequenced them on Illumina’s Hi-Seq 1000 platform. We generated 101 bases long paired-end reads for the parent (30 Million X 2 paired end reads), and recurrent samples (30 Million X 2 paired end reads) from each of the sample. Further, RNA-Seq analytical pipeline as detailed by Trapnell *et al*.^55^ was carried out, mapping these sequence reads to human hg19 genome, with reference gene based annotations from the UCSC’s genome browser (UCSC knowngenes.gtf). FPKM values of all four samples of the same population (i.e SF268, U87MG, PS1 & PS2 parent cells and SF268, U87MG, PS1 & PS2 recurrent cells) were normalized amongst themselves. These normalized FPKM values were used for Principal Component Analysis (PCA) in R (v3.1.0) using *cummeRbund* Bio-conductor package.

### Raman Spectroscopy

#### Sample preparation and spectral acquisition

Equal number of 6 independent generated batch cultures of parent and recurrent cells from U87MG & patient sample 2 and three independent cultures from SF268 and patient sample 1 were harvested and washed with PBS prior to spectra recording. The harvested cell pellet was placed on CaF_2_ window and spectra were acquired using a Raman microprobe system as described earlier^15^. Briefly, this system consists of laser 785 nm (Process Instruments) as an excitation source and HE 785 spectrograph (Horiba-Jobin-Yvon, France) coupled with CCD (Synapse, Horiba-Jobin-Yvon) as dispersion and detection elements, respectively. Optical filtering of unwanted noise, including Rayleigh signals, is accomplished through ‘Superhead’, the other component of the system. Optical fibres were employed to carry the incident light from the excitation source to the sample and also to collect the Raman scattered light from the sample to the detection system. Spectra acquired at excitation wavelength (λex) = 785 nm, laser power = 30 mW, were integrated for 10 seconds and averaged over 6 accumulations. Estimated laser spot size at the cell pellet sample was 5–10 μm. Approximately 5–6 spectra were acquired from each cell pellet. Thus, a total of 30 spectra per group were acquired for each of the parent and recurrent population.

Spectral acquisition of GBM tissues was carried out by a fibre optic Raman probe (In Photonics Inc, Downy St. USA) consisting of an excitation and a collection fibre of diameters 105 and 200 μm, respectively. This commercial Raman probe was coupled to the Raman spectrometer described above. Spectral acquisition parameters were: λex = 785 nm, laser power−80 mW, spectra were integrated for 15 seconds and averaged over 3 accumulations. About 7–8 spectra were recorded for each GBM tissue.

#### Spectral pre-processing and data analysis

The acquired Raman spectra were corrected for CCD response and spectral contaminations from substrate and fibre signals. To remove interference of the slow moving background, first derivatives of spectra (Savitzky-Golay method and window size 3) were computed^15^,^25^,^50^. Spectra were interpolated in the two regions: fingerprint range (700–1800 cm^−1^) and high wavenumber region (2800–3100 cm^−1^) for the cell line study while 1200–1800 cm^−1^ for the GBM tissue study. Interpolated first derivative and vector normalized spectra were then subjected to multivariate unsupervised Principal component analysis (PCA) and supervised Principal component-linear discriminant analysis (PC-LDA). In brief, Principal Component analysis (PCA) is routinely used method for data compression and visualization. It describes data variance by identifying a new set of orthogonal features, called as principal components (PCs) or factors. In LDA, the classification criterion is identified using the scatter measure of within class and between class variance. LDA can be used in conjunction with PCA (PC-LDA) to increase the efficiency of classification. The advantage of doing this is to remove or minimize noise from the data and concentrate on variables important for classification. In our analysis, significant principal components (p < 0.05) were selected as input for LDA. In order to avoid over-fitting of the data, as a thumb rule, total number of factors selected for analysis were less than half the number of the spectra in the smallest group^50^. PC-LDA models were validated by Leave-one-out cross-validation (LOOCV). Leave-one-out cross validation is a type of rotation estimation, a technique used for assessing performance of a predictive model with a hypothetical validation set when an explicit validation set is not available. Algorithms for these analyses were implemented in MATLAB (Mathworks Inc.) based in-house software^56^.

For spectral analysis, average spectra were computed from the background-subtracted spectra prior to derivatization for each class and were baseline-corrected by fitting a fifth order polynomial function. These baseline corrected, smoothed (Savitzky–Golay, 3) and vector-normalized spectra were the used for spectral comparisons. Spectral assignments were performed as per existing literature^38^,^39^.

### Statistical analysis

Two tailed student t-test was applied to the biological data to determine the statistical significance.

## Additional Information

**How to cite this article**: Kaur, E. *et al*. Unique spectral markers discern recurrent Glioblastoma cells from heterogeneous parent population. *Sci. Rep.* **6**, 26538; doi: 10.1038/srep26538 (2016).

## Supplementary Material

Supplementary Information

## Figures and Tables

**Table 1:** PC-LDA Standard model for recurrent and parent cells.

Confusion Matrix								
	PS1 P	PS1 R	PS2 P	PS2 R	SF268 P	SF268 R	U87MG P	U87MG R
PS1 P	17	0	0	0	0	0	0	0
PS1 R	0	16	0	0	0	0	0	0
PS2 P	0	0	16	0	0	0	0	0
PS2 R	1	0	0	14	0	1	0	0
SF268 P	0	0	0	0	20	0	0	0
SF268 R	0	0	0	1	0	18	0	0
U87MG P	0	0	0	0	0	0	18	0
U87MG R	0	0	0	5	0	0	1	16
Leave-one Out cross (LOOCV) confusion matrix								
PS1 P	17	0	0	0	0	0	0	0
PS1 R	0	16	0	0	0	0	0	0
PS2 P	0	0	16	0	0	0	0	0
PS2 R	1	0	0	14	0	1	0	0
SF268 P	0	0	0	0	20	0	0	0
SF268 R	0	0	0	1	0	18	0	0
U87MG P	0	0	0	0	0	0	18	0
U87MG R	0	0	0	5	0	1	1	15
Test prediction								
	**PS1 P**		**PS1 R**		**PS2 P**		**PS2 R**	
PS2 P	0		0		16		0	
PS2 R	1		0		0		14	
U87MG P	0		0		0		0	
U87MG R	0		0		0		5	

**Table 2:** PC-LDA standard model for responding and non-responding GBM tissues.

Confusion matrix		
	Responders	Non-responders
Responders	34	11
Non-responders	3	42
Leave-one Out cross (LOOCV) confusion matrix		
Responders	31	14
Non-responders	3	42

## References

[b1] Ferlay J. . Estimates of worldwide burden of cancer in 2008: GLOBOCAN 2008. Int J Cancer 127, 2893–2917, doi: 10.1002/ijc.25516 (2010). 21351269

[b2] Adamson C. . Glioblastoma multiforme: a review of where we have been and where we are going. Expert Opin Investig Drugs 18, 1061–1083, doi: 10.1517/13543780903052764 (2009). 19555299

[b3] Stupp R. . Radiotherapy plus concomitant and adjuvant temozolomide for glioblastoma. N Engl J Med 352, 987–996, doi: 10.1056/NEJMoa043330 (2005). 15758009

[b4] Inda M. M., Bonavia R. & Seoane J. Glioblastoma multiforme: a look inside its heterogeneous nature. Cancers (Basel) 6, 226–239, doi: 10.3390/cancers6010226 (2014). 24473088 PMC3980595

[b5] Sun S. . Protein alterations associated with temozolomide resistance in subclones of human glioblastoma cell lines. J Neurooncol 107, 89–100, doi: 10.1007/s11060-011-0729-8 (2012). 21979894 PMC3273683

[b6] Hirschmann-Jax C. . A distinct “side population” of cells with high drug efflux capacity in human tumor cells. Proc Natl Acad Sci USA 101, 14228–14233, doi: 10.1073/pnas.0400067101 (2004). 15381773 PMC521140

[b7] Galli R. . Isolation and characterization of tumorigenic, stem-like neural precursors from human glioblastoma. Cancer Res 64, 7011–7021, doi: 10.1158/0008-5472.CAN-04-1364 (2004). 15466194

[b8] Zeppernick F. . Stem Cell Marker CD133 Affects Clinical Outcome in Glioma Patients. Clin Cancer Res 14, 123–129, doi: 10.1158/1078-0432.ccr-07-0932 (2008). 18172261

[b9] Chakravarti A., Chakladar A., Delaney M. A., Latham D. E. & Loeffler J. S. The epidermal growth factor receptor pathway mediates resistance to sequential administration of radiation and chemotherapy in primary human glioblastoma cells in a RAS-dependent manner. Cancer Res 62, 4307–4315 (2002). 12154034

[b10] Chakravarti A. . Survivin enhances radiation resistance in primary human glioblastoma cells via caspase-independent mechanisms. Oncogene 23, 7494–7506, doi: 10.1038/sj.onc.1208049 (2004). 15326475

[b11] Kitange G. J. . Induction of MGMT expression is associated with temozolomide resistance in glioblastoma xenografts. Neuro Oncol 11, 281–291, doi: 10.1215/15228517-2008-090 (2009). 18952979 PMC2718972

[b12] Ye F. . Protective properties of radio-chemoresistant glioblastoma stem cell clones are associated with metabolic adaptation to reduced glucose dependence. PLoS One 8, e80397, doi: 10.1371/journal.pone.0080397 (2013). 24260384 PMC3832364

[b13] Ryzhikova E. . Raman spectroscopy of blood serum for Alzheimer’s disease diagnostics: specificity relative to other types of dementia. J Biophotonics 9999, doi: 10.1002/jbio.201400060 (2014). PMC4575592 25256347

[b14] Ellis D. I., Cowcher D. P., Ashton L., O’Hagan S. & Goodacre R. Illuminating disease and enlightening biomedicine: Raman spectroscopy as a diagnostic tool. Analyst 138, 3871–3884, doi: 10.1039/c3an00698k (2013). 23722248

[b15] Sahu A., Dalal K., Naglot S., Aggarwal P. & Murali Krishna C. Serum based diagnosis of asthma using Raman spectroscopy: an early phase pilot study. PLoS One 8, e78921, doi: 10.1371/journal.pone.0078921 (2013). 24250817 PMC3826756

[b16] Chang V. T. . Quantitative physiology of the precancerous cervix *in vivo* through optical spectroscopy. Neoplasia 11, 325–332 (2009). 19308287 10.1593/neo.81386 PMC2657880

[b17] Yamazaki H., Kaminaka S., Kohda E., Mukai M. & Hamaguchi H. O. The diagnosis of lung cancer using 1064-nm excited near-infrared multichannel Raman spectroscopy. Radiat Med 21, 1–6 (2003). 12801137

[b18] Li S. . Identification and characterization of colorectal cancer using Raman spectroscopy and feature selection techniques. Opt Express 22, 25895–25908, doi: 10.1364/OE.22.025895 (2014). 25401621

[b19] Singh S. P., Deshmukh A., Chaturvedi P. & Murali Krishna C. *In vivo* Raman spectroscopic identification of premalignant lesions in oral buccal mucosa. J Biomed Opt 17, 105002, doi: 10.1117/1.JBO.17.10.105002 (2012). 23223996

[b20] Zhou Y. . Human brain cancer studied by resonance Raman spectroscopy. J Biomed Opt 17, 116021, doi: 10.1117/1.JBO.17.11.116021 (2012). 23154776 PMC3499405

[b21] Evers D., Hendriks B., Lucassen G. & Ruers T. Optical spectroscopy: current advances and future applications in cancer diagnostics and therapy. Future Oncol 8, 307–320, doi: 10.2217/fon.12.15 (2012). 22409466

[b22] Mizuno A. . Near-infrared FT-Raman spectra of the rat brain tissues. Neurosci Lett 141, 47–52 (1992). 1508399 10.1016/0304-3940(92)90331-z

[b23] Mizuno A., Kitajima H., Kawauchi K., Muraishi S. & Ozaki Y. Near-infrared Fourier transform Raman spectroscopic study of human brain tissues and tumours. J Raman Spectrosc 25, doi: 10.1002/jrs.1250250105 (1994).

[b24] Beljebbar A., Dukic S., Amharref N. & Manfait M. *Ex vivo* and *in vivo* diagnosis of C6 glioblastoma development by Raman spectroscopy coupled to a microprobe. Anal Bioanal Chem 398, 477–487, doi: 10.1007/s00216-010-3910-6 (2010). 20577720

[b25] Koljenovic S. . Discriminating vital tumor from necrotic tissue in human glioblastoma tissue samples by Raman spectroscopy. Lab Invest 82, 1265–1277 (2002). 12379761 10.1097/01.lab.0000032545.96931.b8

[b26] Ji M. . Rapid, label-free detection of brain tumors with stimulated Raman scattering microscopy. Sci Transl Med 5, 201ra119, doi: 10.1126/scitranslmed.3005954 (2013). PMC3806096 24005159

[b27] Jermyn M. . Intraoperative brain cancer detection with Raman spectroscopy in humans. Sci Transl Med 7, 274ra219, doi: 10.1126/scitranslmed.aaa2384 (2015). 25673764

[b28] Harder S. J. . A Raman spectroscopic study of cell response to clinical doses of ionizing radiation. Appl Spectrosc 69, 193–204, doi: 10.1366/14-07561 (2015). 25588147

[b29] Devpura S. . Vision 20/20: the role of Raman spectroscopy in early stage cancer detection and feasibility for application in radiation therapy response assessment. Med Phys 41, 050901, doi: 10.1118/1.4870981 (2014). 24784365

[b30] Vidyasagar M. S. . Prediction of radiotherapy response in cervix cancer by Raman spectroscopy: a pilot study. Biopolymers 89, 530–537, doi: 10.1002/bip.20923 (2008). 18189303

[b31] Shaikh R., Vidyasagar M. S. & Krishna C. M. Raman Spectroscopy of Tissues Collected at Different Fractions of Radiation Therapy: Response Assessment to Radiotherapy in Cervix Cancers. J Innov Opt Health Sci 06, 8, doi: 10.1142/S1793545813500144 (2013).

[b32] Yasser M., Shaikh R., Chilakapati M. K. & Teni T. Raman spectroscopic study of radioresistant oral cancer sublines established by fractionated ionizing radiation. PLoS One 9, e97777, doi: 10.1371/journal.pone.0097777 (2014). 24841281 PMC4026477

[b33] Sahu A., Nandakumar N., Sawant S. & Krishna C. M. Recurrence prediction in oral cancers: a serum Raman spectroscopy study. Analyst 140, 2294–2301, doi: 10.1039/c4an01860e (2015). 25619332

[b34] Kaur E. . Radiation-induced homotypic cell fusions of innately resistant glioblastoma cells mediate their sustained survival and recurrence. Carcinogenesis 36, 685–695, doi: 10.1093/carcin/bgv050 (2015). 25863126

[b35] Schröder R., Feisel K. & Ernestus R.-I. Ki-67 Labeling is Correlated with the Time to Recurrence in Primary Glioblastomas. J Neurooncol 56, 127–132, doi: 10.1023/A:1014527929948 (2002). 11995813

[b36] Xie D. . Expression of cytoplasmic and nuclear Survivin in primary and secondary human glioblastoma. Br J Cancer 94, 108–114, doi: 10.1038/sj.bjc.6602904 (2006). 16404364 PMC2361075

[b37] Chakravarti A. . Quantitatively determined survivin expression levels are of prognostic value in human gliomas. J Clin Oncol 20, 1063–1068 (2002). 11844831 10.1200/JCO.2002.20.4.1063

[b38] Parker F. S. Applications of infrared, Raman, and resonance Raman spectroscopy in biochemistry. (Springer Science & Business Media, 1983).

[b39] Movasaghi Z., Rehman S. & Rehman I. U. Raman Spectroscopy of Biological Tissues. Appl Spectrosc Rev 42, 493–541, doi: 10.1080/05704920701551530 (2007).

[b40] Stone N., Kendall C., Smith J., Crow P. & Barr H. Raman spectroscopy for identification of epithelial cancers. Farad Discuss 126, 141–157, doi: 10.1039/B304992B (2004). 14992404

[b41] Stone N., Kendall C., Shepherd N., Crow P. & Barr H. Near-infrared Raman spectroscopy for the classification of epithelial pre-cancers and cancers. J Raman Spectrosc 33, 564–573, doi: 10.1002/jrs.882 (2002).

[b42] Kalkanis S. N. . Raman spectroscopy to distinguish grey matter, necrosis, and glioblastoma multiforme in frozen tissue sections. J Neurooncol 116, 477–485, doi: 10.1007/s11060-013-1326-9 (2014). 24390405

[b43] Kunjachan S., Rychlik B., Storm G., Kiessling F. & Lammers T. Multidrug resistance: Physiological principles and nanomedical solutions. Adv Drug Deliv Rev 65, 1852–1865, doi: 10.1016/j.addr.2013.09.018 (2013). 24120954 PMC3939439

[b44] Gelsomino G. . Omega 3 fatty acids chemosensitize multidrug resistant colon cancer cells by down-regulating cholesterol synthesis and altering detergent resistant membranes composition. Mol Cancer 12, 137, doi: 10.1186/1476-4598-12-137 (2013). 24225025 PMC4225767

[b45] Todor I. N., Lukyanova N. Y. & Chekhun V. F. The lipid content of cisplatin- and doxorubicin-resistant MCF-7 human breast cancer cells. Exp Oncol 34, 97–100 (2012). 23013760

[b46] Liu Y. Y. . A role for ceramide in driving cancer cell resistance to doxorubicin. FASEB J 22, 2541–2551, doi: 10.1096/fj.07-092981 (2008). 18245173

[b47] Liu Y. Y., Hill R. A. & Li Y. T. Ceramide glycosylation catalyzed by glucosylceramide synthase and cancer drug resistance. Adv Cancer Res 117, 59–89, doi: 10.1016/B978-0-12-394274-6.00003-0 (2013). 23290777 PMC4051614

[b48] Legler J. M. . Cancer surveillance series [corrected]: brain and other central nervous system cancers: recent trends in incidence and mortality. J Natl Cancer Inst 91, 1382–1390 (1999). 10451443 10.1093/jnci/91.16.1382

[b49] Walid M. S. Prognostic factors for long-term survival after glioblastoma. Perm J 12, 45–48 (2008). 21339920 10.7812/tpp/08-027 PMC3037140

[b50] Crow P. . The use of Raman spectroscopy to differentiate between different prostatic adenocarcinoma cell lines. Br J Cancer 92, 2166–2170, doi: 10.1038/sj.bjc.6602638 (2005). 15928665 PMC2361812

[b51] Sahu A. . Raman spectroscopy and cytopathology of oral exfoliated cells for oral cancer diagnosis. Analytical Methods 7, 7548–7559, doi: 10.1039/C5AY00954E (2015).

[b52] Rubina S., Amita M., Kedar D., Bharat R. & M.C. K. Raman spectroscopic study on classification of cervical cell specimens. Vib Spectrosc 68, 115–121, doi: 10.1016/j.vibspec.2013.06.002 (2013).

[b53] Krishna M. C. . Micro-Raman spectroscopy of mixed cancer cell populations. Vib Spectrosc 38, 95–100, doi: 10.1016/j.vibspec.2005.02.018 (2005).

[b54] Murali Krishna C. . Characterisation of uterine sarcoma cell lines exhibiting MDR phenotype by vibrational spectroscopy. Biochim Biophys Acta 1726, 160–167, doi: 10.1016/j.bbagen.2005.08.006 (2005). 16169664

[b55] Trapnell C. . Differential gene and transcript expression analysis of RNA-seq experiments with TopHat and Cufflinks. Nat Protoc 7, 562–578, doi: 10.1038/nprot.2012.016 (2012). 22383036 PMC3334321

[b56] Ghanate A. D., Kothiwale S., Singh S. P., Bertrand D. & Krishna C. M. Comparative evaluation of spectroscopic models using different multivariate statistical tools in a multicancer scenario. J Biomed Opt 16, 025003, doi: 10.1117/1.3548303 (2011). 21361683

