# Supplementary material for: Unique spectral markers discern recurrent Glioblastoma cells from heterogeneous parent population
Source: Sci Rep. 2016 May 25;6:26538. doi: 10.1038/srep26538 (PMC4879554; doi:10.1038/srep26538)
Supplement: Supplementary Information [file srep26538-s1.pdf]

Unique spectral markers discern recurrent Glioblastoma cells from heterogenous parent population

Ekjot Kaur, Aditi Sahu, Arti Nilesh Adhav, Jacinth Rajendra, Rohan Chaubal, Nilesh Gardi, Amit Dutt, Aliasgar Moiyadi, C. Murali Krishna*, Shilpee Dutt*

*Corresponding authors: Shilpee Dutt, email:sdutt@actrec.gov.in
Murali Krishna Chilakapati, email:mchilakapati@actrec.gov.in

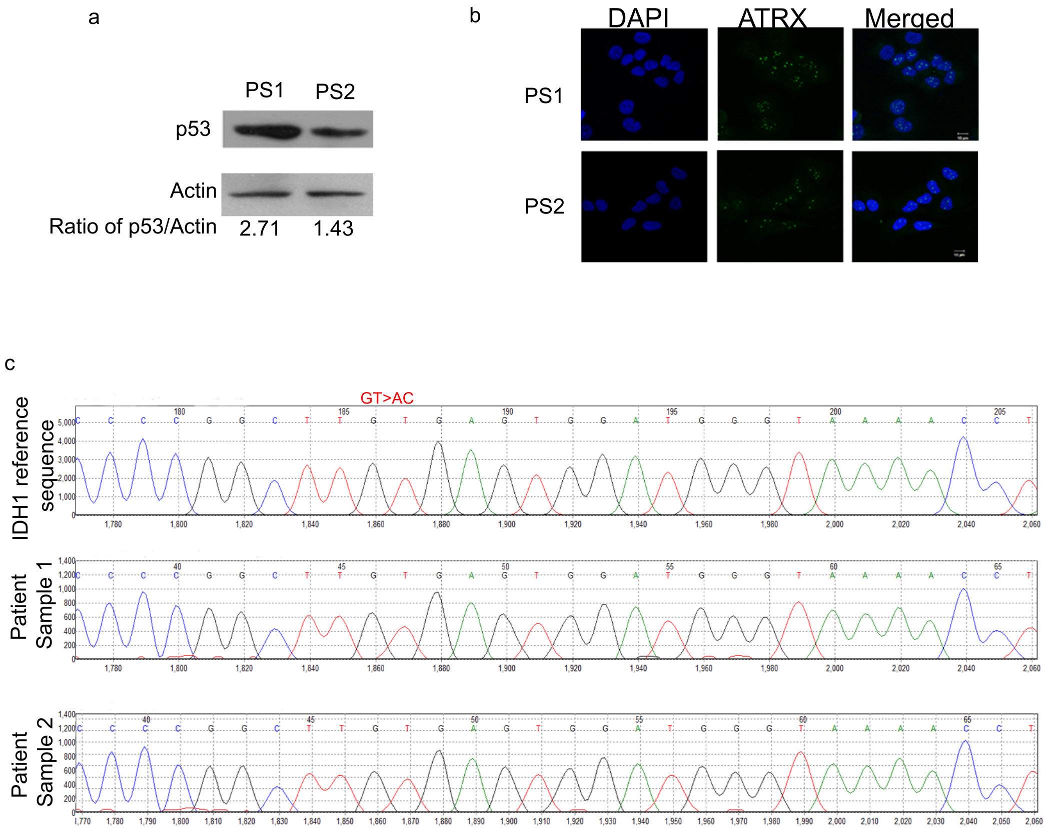

Supplementary Figure 1: Primary cultures do not harbour IDH1 mutation and show p53 and ATRX protein expression.
a) Western blot analysis for p53 and actin for patient samples 1 and patient sample 2 (PS1 and PS2 respectively).
b) Representative images for ATRX expression in PS1 and PS2.
c) Sanger sequencing chromatogram depicting the IDH1 R132H traces in Patient sample 1 and 2. Reference sequence trace is shown in upper panel and the mutated bases are shown in red.

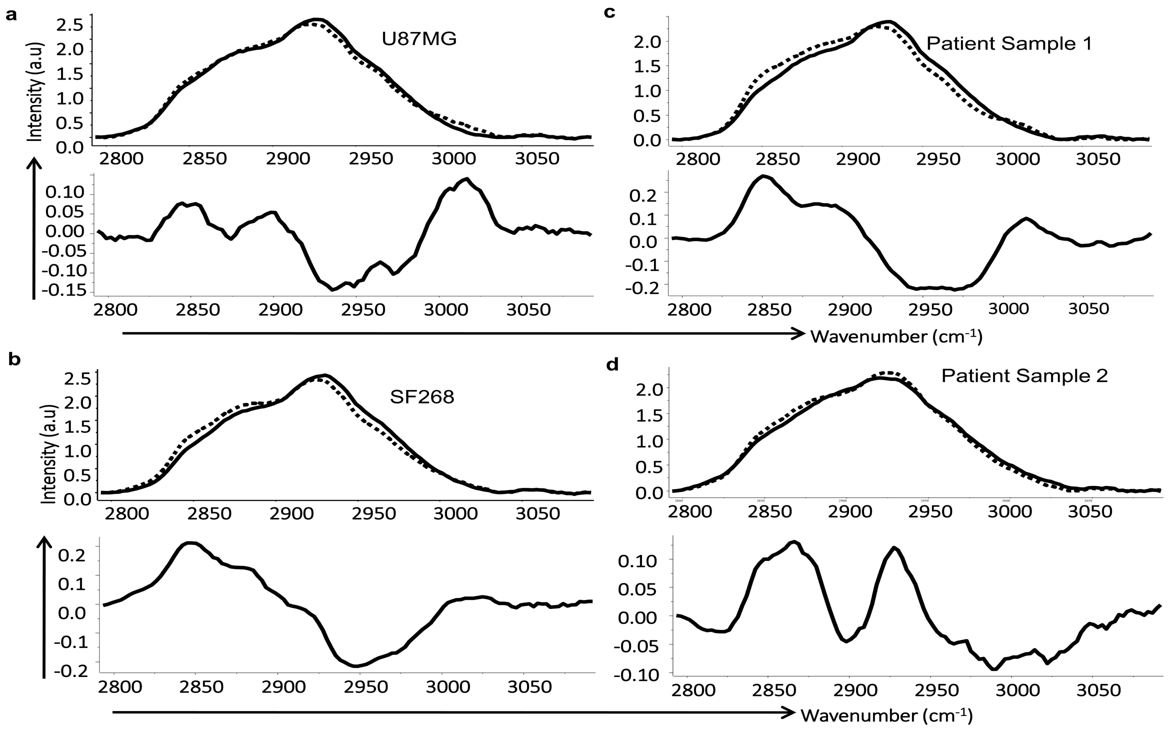

Supplementary Figure 2a-d: Mean and difference spectra from parent and recurrent population of the high wavenumber region indicate higher lipid content in the recurrent cells compared to the parent cells. Dotted lines represent the spectra from recurrent cells while solid lines represent spectra from parent cells.
